# Death, dying and bereavement care during COVID-19: Creativity in hospital social work practice

**DOI:** 10.1177/1473325020981074

**Published:** 2021-03

**Authors:** Mim Fox, Joanna McIlveen, Elisabeth Murphy

**Affiliations:** Faculty of Social Sciences, School of Health & Society, University of Wollongong, Liverpool, Australia; St George Hospital, Kogarah, Australia; Wollongong Hospital, Wollongong, Australia

**Keywords:** Social work practice, hospitals, bereavement, bereavement care, viewings, COVID-19

## Abstract

Bereavement support and conducting viewings for grieving family members are commonplace activities for social workers in the acute hospital setting, however the risks that COVID-19 has brought to the social work role in bereavement care has necessitated the exploration of creative alternatives. Social workers are acutely aware of the complicating factors when bereavement support is inadequately provided, let alone absent, and with the aid of technology and both individual advocacy, social workers have been able to continue to focus on the needs of the most vulnerable in the hospital system. By drawing on reflective journaling and verbal reflective discussions amongst the authors, this article discusses bereavement support and the facilitation of viewings as clinical areas in which hospital social work has been observed adapting practice creatively throughout the pandemic.

## Introduction

The relationship between social work practice, social work research, public health policy and healthcare outcomes has previously been argued for (Ruth and Marshall, 2017), as has the need for a coordinated psychological response for those in an acute care setting where COVID-19 wards have been established (Duan and Zhu, 2020). The potential for reactive guidelines and procedures in hospitals which complicate the grieving process for both affected individuals and their loved ones has been articulated (Wallace et al., 2020), yet for social workers who work every day in these essential institutions, they are needing to continue to provide a social work service that is often counter-intuitive to this impact. Although death and dying is a constant feature of hospital social work in Australia, COVID-19 has firmly secured the social work profession as fundamental in supporting those most vulnerable in our system. The current “second wave” of COVID-19 infection rates in Australia has brought with it similar resourcing concerns that have been seen internationally, with an increase in temporary testing sites and hospital admissions. A noticeable gap when the pandemic began was the lack of practice principles and resources to support hospital social work staff. The authors of this article have all practised in this field and through reflective journaling and discussions held regularly amongst hospital colleagues, a picture of change has emerged.

## The witnessing of change in hospital social work practice

Since early March 2020, Australian health workers have had to be extremely adaptable to the changing circumstances with which the COVID-19 pandemic presented. Social workers have continued to work in acute hospital settings, often with greater numbers being recruited or redeployed to COVID-19 wards. This led to a new daily experience of the hospital, complete with safety measures in place. The changes evolved on a daily basis and adapting to these changes required flexibility. All visitors and health care workers are still today screened at main entrances of facilities to minimise risk of COVID- 19 transmission.

Visitor policies were revised, and visitor attendance was discouraged in an attempt to reduce foot traffic through clinical spaces. Delivering any service to COVID-19 positive or suspected patient has involved the strict use of personal protective equipment (PPE). No visitors have been allowed to visit these patients, unless in exceptional circumstances, and all patients and staff have had to comply with physical distancing, hygiene, infection prevention and control protocols (Health Protection NSW, 2020a).

We, the authors, have together critically questioned the efficacy of practice principles and modes of intervention that have historically been used in bereavement support. We discussed the lived experience of sitting closely with grieving individuals and families, often in close proximity to bodily fluids, often involving a supportive embrace or therapeutic touch. How do we now hold space for a grieving person if we cannot sit side by side and cannot reach out a comforting hand? How do we demonstrate empathy when the PPE you are mandated to wear shows very little of your face and muffles your voice? Finally, how do we facilitate continuing bonds for a grieving person when the policies do not allow them to visit with their loved ones before or after death?

## Social workers and reflective ways of knowing

Critical reflection in social work practice is described as a process of self-evaluation whereby external contexts influence the social worker’s practice and values (Yip, 2006). Critical reflection has become closely linked with reflective practice and reflexivity (Watts, 2019), whereby the social worker is evaluating their own practices and performance through their internal thoughts and feelings. Practice wisdom is seen as the source of critical reflection, the coming together of complexity and enquiry, to form professional decision making (Cheung, 2016). Storytelling and reflective journaling are used regularly to debrief complex practice situations or encounters (Wilder Craig, 2007), and to engage with practice wisdom (Weike, 2000). For Australian social work to decolonise our practice there is an imperative on us to engage with ways of knowing and understanding that are not strictly science or logic based, but grounded in lived experience (Terare and Rawsthorne, 2020).

Journaling, reflexive discussion and storytelling is utilised here as a qualitative method of enquiry (Symon, 2004), with the authors together having participated in a co-analysis of the written and verbal reflections. This was undertaken recognising the frequency of themes raised in collegial discussions, and the lived experience of the grieving individuals we interact with. Criticism of journaling as data collection are that it can be subjective and self-indulgent in its internal perspective (Denzin, 2006). However, the work that social workers do is often invisible to the untrained eye, and it is for this reason that social workers engage in subjective practice narration. With this in mind the reflections in this article are presented with the aim of documenting practitioner creativity at a time when creativity is difficult and reflective space in itself, an indulgence.

## Hospital social work practice

Social workers who practice in hospitals are familiar with death and dying and daily call on a variety of practice interventions (Lewis and Fox, 2019). These social workers who work primarily in bereavement support engage in companioning as a primary intervention; bearing witness to, and regularly immersing themselves in, the acute grief of others; holding space and validating the existence of, and expression of, a grief response (Wolfelt, 2009). Social workers partner in bereavement support regularly with medical, nursing and pastoral care colleagues, whilst continuously assessing family dynamics, coping and bereavement risk (Duffy and Healy, 2011). Throughout all of this hospital social workers are acutely aware that they have been privileged to stand with these grieving individuals, that their intervention is time bound and context specific.

## Hospital social work with death and dying during COVID-19

Due to the sudden impact of COVID-19 across the globe what we know about social work practice during this time has come largely from personal narratives or opinion pieces, such as editorials and published correspondence. Experiences that reflect the disproportionate effect of COVID-19 on minority groups (Rajan-Rankin, 2020) and the impact of social injustice have been shared (O’Leary and Tsui, 2020). The disruption of the social process of death has been highlighted, as well as the limitations on visitors for those dying and the lack of grief rituals for those affected (Ingravallo, 2020). Technology has been seen as useful in bereavement, but also has emerged as the modern-day societal divider (Walter-McCabe, 2020). Throughout there is an assertion that our global social work response be values driven (McGowan, 2020). We acknowledge that given the relatively low transmission and infection rate in Australia we have had the luxury of reflecting collectively, discussing and debating the parameters of our practice. We are acutely aware that this has not been the case for many of our international colleagues. In light of this there are two areas of grief and bereavement work in the acute hospital setting to highlight that demonstrate the complexity of the practice decisions that are now being made, as well as the creativity of the social workers working in this space.

## Bereavement support

In the hospital setting bereavement support is largely conducted by social workers with a grieving individual or family face to face, in whatever space is available ranging from bedside to carpark. Since the pandemic began social workers have had to adapt their bereavement practices in line with public health restrictions and limited visiting. Practically there has been significant increase in telephone work, and virtual online work, as many families are unable to visit. Social workers have responded to these limitations by ensuring the environment is as soothing as possible for the patient by prioritising their cultural and spiritual needs, particularly given that spiritual leaders and pastoral care also cannot attend. As the conduit between family and the dying patient social workers enable connection of patient and family through virtual bedside visits and the recording of final messages. An area of practice that has emerged creatively and spontaneously in this period has been that of memory making, previously the domain of pregnancy loss or the death of a child (Thornton et al., 2020). Ordinarily, social workers spend time with patients and families, offering them the opportunity to co-create memories through crafting hand or foot prints, locks of hair and photographs as a means of connection and ongoing bond. Since COVID-19, never before have social work had to work so closely with infection control experts to meet the needs of all grieving families whilst maintaining personal safety. Creative practices have included facilitating connection through letters, photos, hand-drawn pictures and music. These mementos have been given to the patient facing the end of their life as a means of comfort and have travelled with them during their chosen death rituals. One social worker recollected an older patient dying from COVID-19 in the Intensive Care Unit (ICU) who was unable to be visited by his family as they were also in isolation. The social worker identified it as important that the grand daughters were able to say goodbye. The social worker explored alternate ways of connection, resulting in the grand daughters writing letters to, and drawing pictures for, their dying grandfather. The social worker printed them out and facilitated the ICU nurse to read and show them to the patient whilst he was dying. Social work and nursing staff worked together to ensure the letters then travelled with the patient to the mortuary, and then to funeral home.

## Viewings

In normal times a hospital social worker would facilitate viewings and opportunities for families to say goodbye by the bedside or in the hospital mortuary. We know from the literature on grief that being able to spend time with the dead facilitates strong grief connections and continuing bonds, and social workers are instrumental in facilitating this process. If the patient is COVID-19 confirmed or suspected, the possibility for viewing is rare so facilitating continuing bonds has taken on a new life. The NSW Health guidelines (Health Protection NSW, 2020b) focus solely on infection control with an emphasis that if relatives are visiting the deceased person they should not touch the body and physical distancing is maintained. Terminology such as “double bagging” whereby the body is placed in two bags and mandated to remain closed now exists. It has fallen to social work to explain this process tactfully, outlining the risks as to why families can’t view the body. Where able, social workers have advocated for family members to be assisted to wear full PPE to view their loved one’s body, or for double bagging to happen slightly later (sometimes minutes later) to give families time to come into the hospital to say goodbye from behind a glass window. In an attempt to facilitate some version of a viewing, social workers have also been observed working individually to support families with viewings, including facilitating “virtual viewings” on a smart device.

## Moving forward

Globally, COVID-19 has demonstrated that death, dying and bereavement is a universal phenomenon and that our acute hospitals play a vital role in establishing norms for death practice and care for the grieving. During this time, hospital social workers have responded to the needs of the bereaved compassionately and courageously. At a time of heightened vulnerability across our community, the practice creativity from social workers is now more important than ever. Social work tasks such as providing bereavement support or facilitating family viewings are often invisible in the fast-paced acute hospital setting, they are not considered life or death activities as are the tasks which engage our multidisciplinary colleagues. That being said, social workers in hospitals deal largely in both life and death and are acutely aware of the impact that complicated grief can bring. In Australia our work has been limited to affected individuals. The global grief however is collective, and bereavement continues to be universal. So too is the creative social work response.
